# Intratumoral habitat and peritumor radiomics for progression risk stratification of patients with soft tissue sarcoma: a multicenter study

**DOI:** 10.3389/fonc.2025.1619704

**Published:** 2026-01-19

**Authors:** Hao-Yu Liang, Chuan-ping Gao, Meng Zhang, Shi-Feng Yang, Feng Hou, Li-Sha Duan, Yong-Hua Huang, Chen-Cui Huang, Jing-Xu Xu, Da-Peng Hao, He-Xiang Wang

**Affiliations:** 1 Department of Radiology, The Affiliated Hospital of Qingdao University, Qingdao, China; 2 Department of Radiology and Institute of Medical Functional and Molecular Imaging, Huashan Hospital, Fudan University, Shanghai, China; 3 Department of Radiology, Shandong Provincial Hospital Affiliated to Shandong First Medical University, Jinan, China; 4 Department of Pathology, The Affiliated Hospital of Qingdao University, Qingdao, China; 5 Department of Radiology, The Third Hospital of Hebei Medical University, Shijiazhuang, China; 6 Department of Radiology, The Puyang Oilfield General Hospital, Puyang, Henan, China; 7 Department of Research Collaboration, Research and Development (R&D) center, Beijing Deepwise & League of Philosophy Doctor (PHD)Technology Co., Ltd, Beijing, China

**Keywords:** habitat imaging, magnetic resonance imaging, progression, radiomics nomogram, soft tissue sarcoma

## Abstract

**Objective:**

To establish and validate a radiomics nomogram that incorporated tumor habitat and peritumor features to predict tumor progression in patients with soft tissue sarcoma (STS).

**Methods:**

MRI data (fat-suppressed T2-weighted and contrast-enhanced fat-suppressed T1-weighted images) from 148 STS patients treated in four institutions were retrospectively enrolled. Patients were divided into a training cohort (n = 108) and validation cohort (n = 40). K-means clustering was applied to split intratumoral voxels into three habitats according to signal intensity values. A large number of radiomics features were extracted from numerous tumor-associated regions (tumor lesion, peritumor, tumor expansion, and intratumoral habitats) to construct a series of radiomics signatures. A nomogram integrating clinical predictors and radiomics signature was established and its value for predicting progression was validated.

**Results:**

The nomogram yielded superior prediction performance and less predictive error in the validation cohort (C-index, 0.777; median area under the receiver operating characteristic curve, 0.808; integrated Brier score, 0.135). When patients were stratified according to risk of progression (low and high) based on the nomogram in both the training and validation cohorts, Kaplan–Meier survival analysis demonstrated significant differences in progression-free survival between the groups. In addition, it could attach incremental value to histopathological grade system in progression risk evaluation.

**Conclusion:**

A nomogram based on intratumoral habitat and peritumor radiomics predicts tumor progression in STS patients and stratifies them according to risk of progression.

## Introduction

Soft tissue sarcomas (STSs) are histologically heterogeneous and account for less than 1% of all malignant tumors (1). Radical resection is the standard treatment for patient with localized disease. Even after resection, the prognosis is poor, as reported recurrence rates range from 33% to 50% (2, 3) and the rate of distant metastasis is approximately 46% (4). Early identification of patients with a high risk of recurrence or metastasis after surgical resection to enable optimal use of standard or intensified neoadjuvant chemoradiotherapy might improve outcomes. This would require formulation of an accurate model for risk stratification in STS patients.

Despite their limitations, the TNM, Fédération Nationale des Centres de Lutte Contre Le Cancer (FNCLCC), and National Cancer Institute (NCI) staging systems are commonly used to guide STS prognostication and treatment (5). Several statistical models based on clinical and pathological data have been constructed and examined in previous studies to predict outcomes in STS patients (5–7). However, these models were based on low-dimensional clinical information and overlooked massive high-dimensional imaging characteristics. Therefore, their performance and generalized applicability are controversial (5, 8).

Radiomics extracts more detailed imaging features than traditional visual interpretation and can provide more data for clinical decision making (9). Radiomics-based models constructed using carefully screened features have the potential to predict STS outcomes (8, 10–13). In previous studies, radiomics data of tumor regions were analyzed as a whole and neglected intratumoral subregions (tumor habitats) with similar radiological phenotypes (14). Aggressive habitats might be crucial for tumor prognosis determination (14, 15). Several studies have demonstrated that tumor habitat analysis has high value in predicting tumor outcomes, both alone (16, 17) and in combination with radiomics analysis (18). Previous studies mainly concentrated on evaluation of the primary tumor and overlooked subtle changes in the peritumoral microenvironment (19, 20). However, the peritumoral microenvironment can explain aggressive biological behavior (21). Therefore, both tumor habitat and peritumoral environment should be evaluated to depict a tumor’s behavior and potential for invasion (14, 22).

This study aimed to establish and validate a radiomics nomogram that incorporated tumor habitat and peritumoral features to predict progression-free-survival (PFS) in patients with STS. We hypothesized that such a nomogram would show enhanced prognostic value.

## Materials and methods

### Patients

The study was approved by the review boards of all participating institutions. The requirement for written informed consent was waived.

We reviewed preoperative MRI data of 309 patients who underwent resection of STS from January 2007 to July 2022 in one of four participating hospitals. A diagnosis of STS was confirmed histopathologically in all. Patients were included if: (i) they had integrated medical data; (ii) STS was confirmed pathologically (with immunohistochemical examination); and (iii) MRI examination was performed within 2 weeks before surgery or preoperative neoadjuvant radiotherapy/chemotherapy, and included FS-T2WI and CE-T1WI. Patients were excluded if medical or imaging data was inadequate or imaging was of poor quality (signal-to-noise ratio<1.0). We also excluded those with a second malignancy and patients who lacked follow-up data.

**Figure 1:**
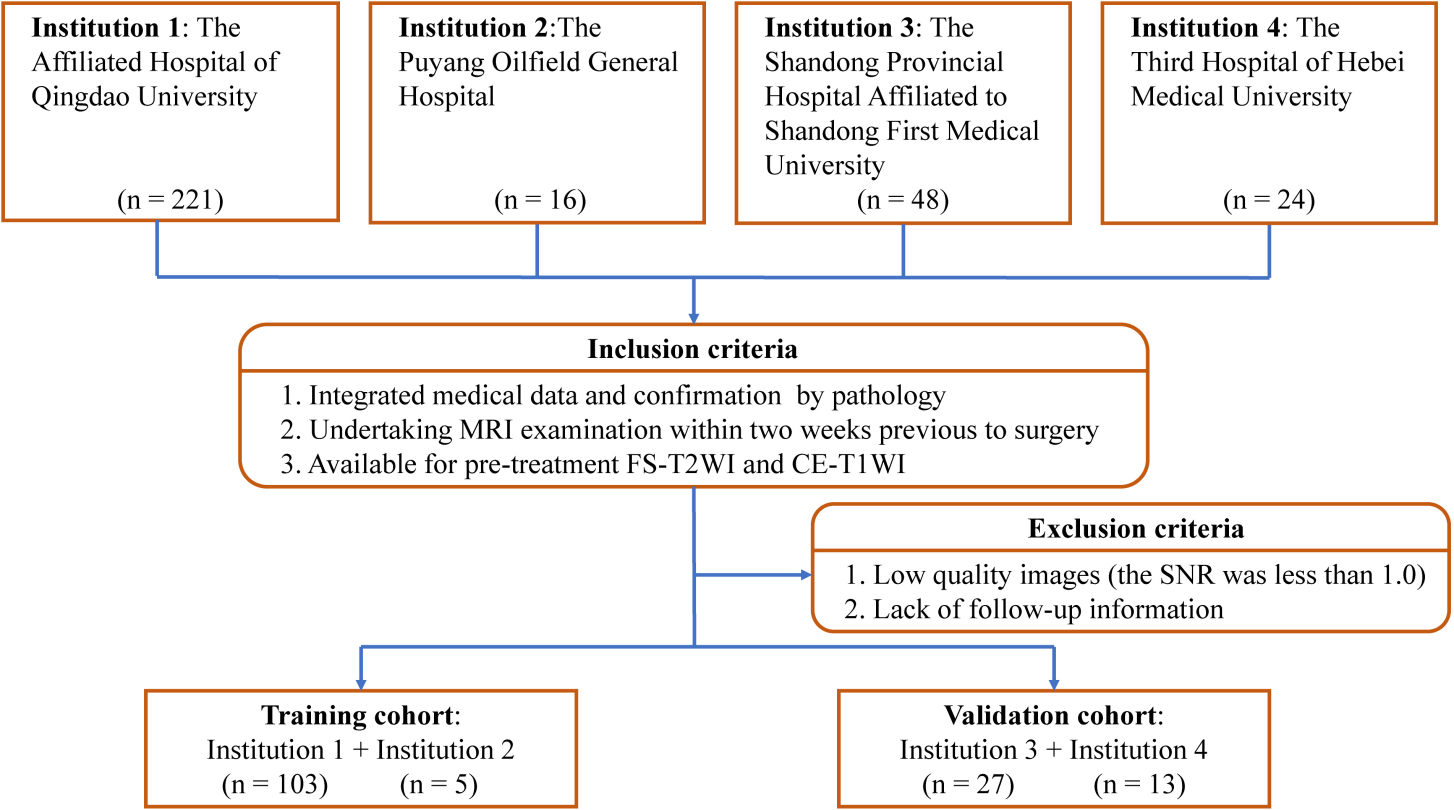
Study inclusion and exclusion criteria. FS-T2WI, fat-suppressed T2-weighted imaging; CE-T1WI, contrast enhanced fat-suppressed T1-weighted imaging; SNR, signal-to-noise ratio.

After applying criteria, 148 patients (average age ± standard deviation, 54 years ± 17) were included for analysis. The training cohort comprised 108 patients from the Affiliated Hospital of Qingdao University and the Puyang Oilfield General Hospital. The validation cohort comprised 40 patients from the Shandong Provincial Hospital Affiliated to Shandong First Medical University and the Third Hospital of Hebei Medical University. The process of patient enrollment is shown in **Figure 1**. The pathological findings are shown in **Supplementary Table S1**.

PFS was defined as the time from surgery to local recurrence, detection of new distant metastases on imaging, death, or last follow-up. Follow-up surveys were conducted every 3 to 6 months for the first 2 years after surgery and every 6 months thereafter. The censoring date was set as December 17, 2022.

### MRI protocol

MRI included axial fat-suppressed T2-weighted imaging (FS-T2WI) and axial contrast-enhanced fat-suppressed T1-weighted imaging (CE-T1WI). Scans were performed using the following scanners: HDx 1.5 T/3.0 T (GE Healthcare, Chicago, IL, USA), Magnetom Skyra 3.0 T (Siemens, Munich, Germany), Achieva 1.5 T (Philips Healthcare, Amsterdam, Netherlands), and Prisma (Siemens). Scanner parameters are listed in **Supplementary Table S2**.

### Clinical data collection and semantic MRI evaluation

Twenty characteristics were collected from among the clinical baseline information, postoperative histopathological indicators and semantic MRI features (**Supplementary A1**).

### Image preprocessing and lesion segmentation

**Figure 2:**
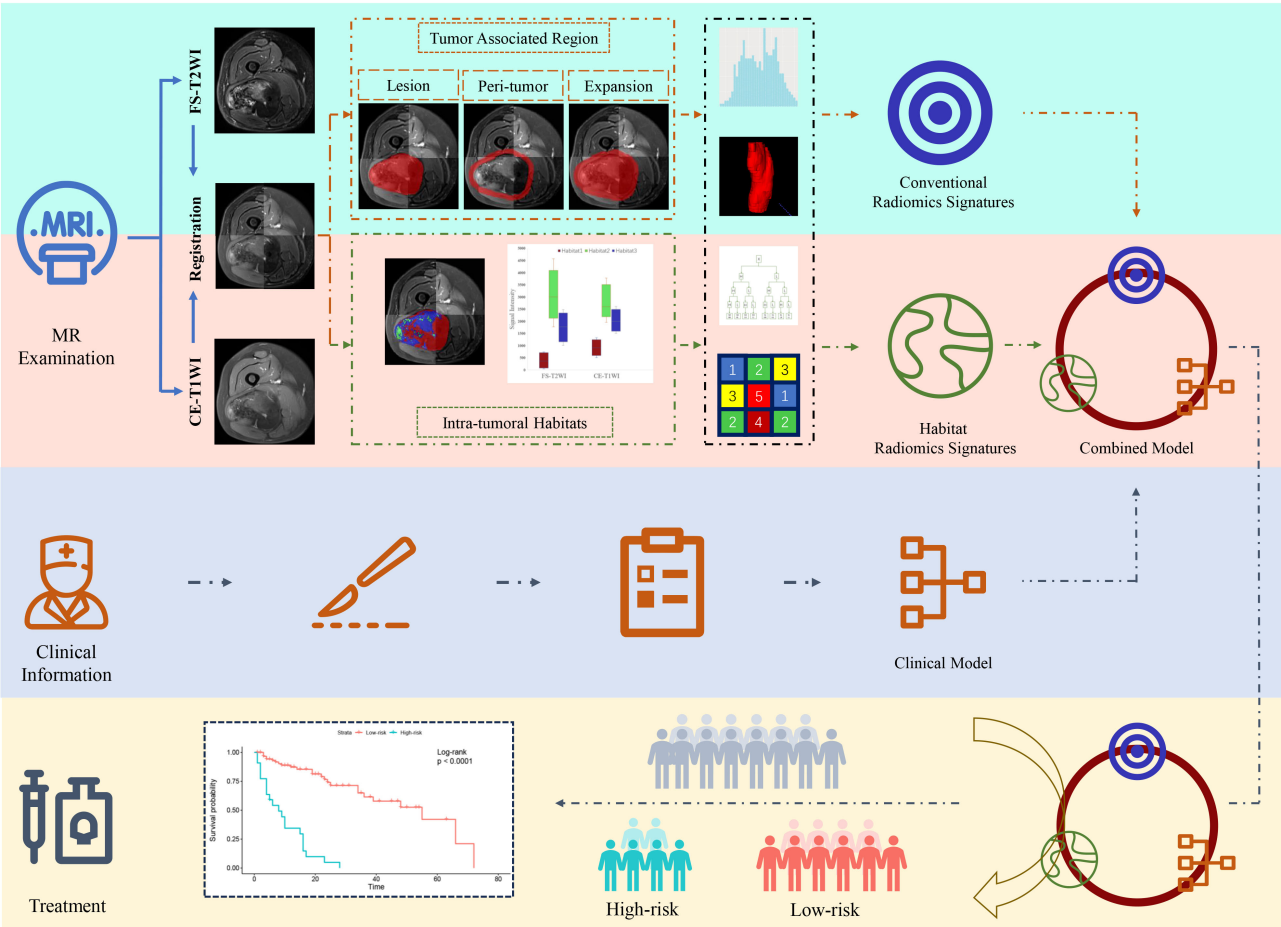
Flowchart of radiomics analysis.

The study flowchart is shown in **Figure 2**. Image preprocessing and segmentation of tumor-associated regions were performed as a four-step procedure which included image registration, N4-bias-field-correction, tumor-associated region segmentation, and spatial resampling (**Supplementary A2**).

Prior to habitat analysis of tumor regions, signal intensity on the FS-T2WI and CE-T1WI images was normalized using the histogram intensity normalization method in Python (23). After applying the K-means clustering module, the voxels in FS-T2WI and CE-T1WI images were aggregated after normalization into three clusters standing for functionally coherent tumor subregions. Two distinct signal intensity maps of the FS-T2WI and CE-T1WI sequences defined the clusters and separated the whole tumor region into 3 intratumoral habitats: habitat 1, a low-enhancing solid subregion with low CE-T1WI and FS-T2WI signal intensity; habitat 2, an enhancing viable subregion with high CE-T1WI and FS-T2WI signal intensity; and habitat 3, a hypoactive subregion with s medium CE-T1WI and FS-T2WI signal intensity.

### Radiomics feature extraction

Feature extraction was processed using PyRadiomics in Python. For the tumor region, peritumoral, and tumoral expansion masks, 1906 conventional radiomics features (containing first-order, shape, textural, and wavelet features) were extracted from each sequence. For the three intratumoral habitats, 93 radiomics features (containing first-order and textural features) were extracted. In addition, voxel number and voxel fraction of each habitat for every patient were recorded as baseline habitat features.

### Standardization and normalization of features

Radiomics features were standardized using combat compensation methodology, which can reduce technical inconsistencies resulting from different scanner protocols (24). Then, they were normalized into a *Z*-score referring to their mean value and standard deviation.

### Progression predictive survival signature determination

The process of survival signatures determination was detailed in **Supplementary A3**. In total, 18 signatures of three sets were built: conventional radiomics signature set, habitat baseline signature set, and habitat radiomics signature set (**Supplementary Table S3**).

The radiomics progression risk score (RPRS) of the best performing radiomics signature was calculated using the following formula:


$$RPRS=\sum_{i=0}^{N}C_{i}\times V_{i}$$

where N is the number of features enrolled into the signature, *V*_i_ is the value of the i^th^ feature, and *C*_i_ is the regression coefficient in the signature.

### Survival model development and validation

The process of clinical model and nomogram construction was detailed in **Supplementary A4**.

### Statistics

Statistical analyses were performed using R software version 4.1.0 (The R Foundation, Vienna, Austria). Continuous data were compared using the *t*-test. Categorical data were compared using the chi-square or Fisher exact test as appropriate. For survival signatures and models, predictive performance was evaluated using the concordance index (C-index) and receiver operating characteristic curve analysis (25). Prediction errors were estimated using the integrated Brier score (IBS). The IBS was evaluated using the “Boot632plus” splitting method (26). Calibration curves and decision curve analysis were used to assess model fitting, clinical reliability, and practicability. Patients were divided into subgroups with different risks of progression based on optimal cut-off values that were determined using X-tile software version 3.6.1 (Yale University School of Medicine, New Haven, CT, USA) (27). PFS was estimated using the Kaplan–Meier method and compared using the log-rank test. Areas under the ROC curve (AUCs) were compared using the DeLong test. P<0.05 was considered significant.

## Results

Median PFS overall 148 patients was 12.5 months (range, 1–88). Mean PFS in patients who experienced STS progression and those who did not was 11 months and 13 months, respectively. Patients from the training and validation cohorts had similar baseline characteristics except for age, FNCLCC grade, NCI grade, American Joint Committee on Cancer (AJCC) stage, histopathological grade, depth, heterogeneous signal intensity on T2WI, radiotherapy, chemotherapy, and tumor location (**Table 1**).

**Table 1:** Patient baseline characteristics.

		Training cohort	Validation cohort	*P*
No. of patients		108	40	
Prognosis	None-progression	67 (45.3)	23 (15.5)	0.616
	Progression	41 (27.7)	17 (11.5)	
PFS (month) *		11.5 [5, 21.5]	23 [9, 46.5]	0.001
Clinical baseline information				
Age (year) #		56 ± 16	28 ± 21	0.002
Gender	Male	57 (38.5)	26 (17.5)	0.183
	Female	51 (34.5)	14 (9.5)	
Postoperative histopathological indicators				
FNCLCC	I	16 (10.8)	16 (10.8)	0.001
	II	32 (21.6)	11 (7.4)	
	III	60 (40.5)	13 (8.8)	
NCI	I	15 (10.1)	15 (10.1)	0.002
	II	35 (23.6)	14 (9.5)	
	III	58 (39.2)	11 (7.4)	
AJCC	I	18 (12.2)	13 (8.8)	0.028
	II	13 (8.8)	9 (6.1)	
	III	61 (41.2)	13 (8.8)	
	IV	16 (10.8)	5 (3.4)	
Histopathological grade	Low	16 (10.8)	16 (10.8)	0.001
	High	92 (62.2)	24 (16.2)	
Semantic MRI features				
Number	Solitary	85 (57.4)	28 (18.9)	0.268
	Multiple	23 (15.5)	12 (8.1)	
Depth	Deep	34 (23.0)	23 (15.5)	0.004
	Superficial	74 (50.0)	17 (11.5)	
Heterogeneous SI at FS-T2WI	<50%	64 (43.2)	12 (8.1)	0.002
	≥50%	44 (29.7)	28 (18.9)	
Tumor volume with MRI signal compatible with necrosis	0	31 (20.9)	7 (4.7)	0.276
	1%–50%	57 (38.5)	22 (14.9)	
	>50%	20 (13.5)	11 (7.4)	
Margin definitions at CE-T1WI	Well-defined≥90%	47 (31.8)	15 (10.1)	0.15
	Well-defined50%-90%	50 (33.8)	16 (10.8)	
	Well-defined<50%	11 (7.4)	9 (6.1)	
Peritumoral edema	No	22 (14.9)	8 (5.4)	0.602
	Limited	76 (51.4)	26 (17.6)	
	Extensive	10 (6.8)	6 (4.1)	
Peritumoral enhancement	+	54 (36.5)	14 (9.5)	0.104
	–	54 (36.5)	26 (17.6)	
T-stage	1	22 (14.9)	6 (4.1)	0.422
	2	34 (23.0)	18 (12.2)	
	3	21 (14.2)	8 (5.4)	
	4	31 (20.9)	8 (5.4)	
N-stage	0	89 (60.1)	34 (23.0)	0.709
	1	19 (12.8)	6 (4.1)	
M-stage	0	87 (58.8)	34 (23.0)	0.534
	1	21 (14.2)	6 (4.1)	
Surgical margins	R0·	89 (60.1)	36 (24.3)	0.258
	R1	19 (12.8)	4 (2.7)	
Radiotherapy	No	77 (52.0)	12 (8.1)	0.001
	Adjuvant	31 (20.9)	28 (18.9)	
Chemotherapy	No	73 (49.3)	17 (11.5)	0.005
	Adjuvant	35 (23.6)	23 (15.5)	
Location	Limbs	80 (54.1)	17 (11.5)	0.001
	Trunk wall	6 (4.1)	6 (4.1)	
	Head and neck	11 (7.4)	3 (2.0)	
	Internal trunk	11 (7.4)	14 (9.5)	

Data are numbers of participants; data in parentheses are percentages.

PFS, progression-free-survival; FNCLCC, Fédération Nationale des Centres de Lutte Contre le Cancer; NCI, National Cancer Institute; AJCC, American Joint Committee on Cancer; SI, signal intensity; FS-T2WI, fat-suppressed T2-weighted imaging; CE-T1WI, contrast-enhanced fat-suppressed T1-weighted imaging.

*Data are median [inter-quartile range]; #Data are means ± standard deviation.

### Habitat analysis and radiomics signature development

**Figure 3:**
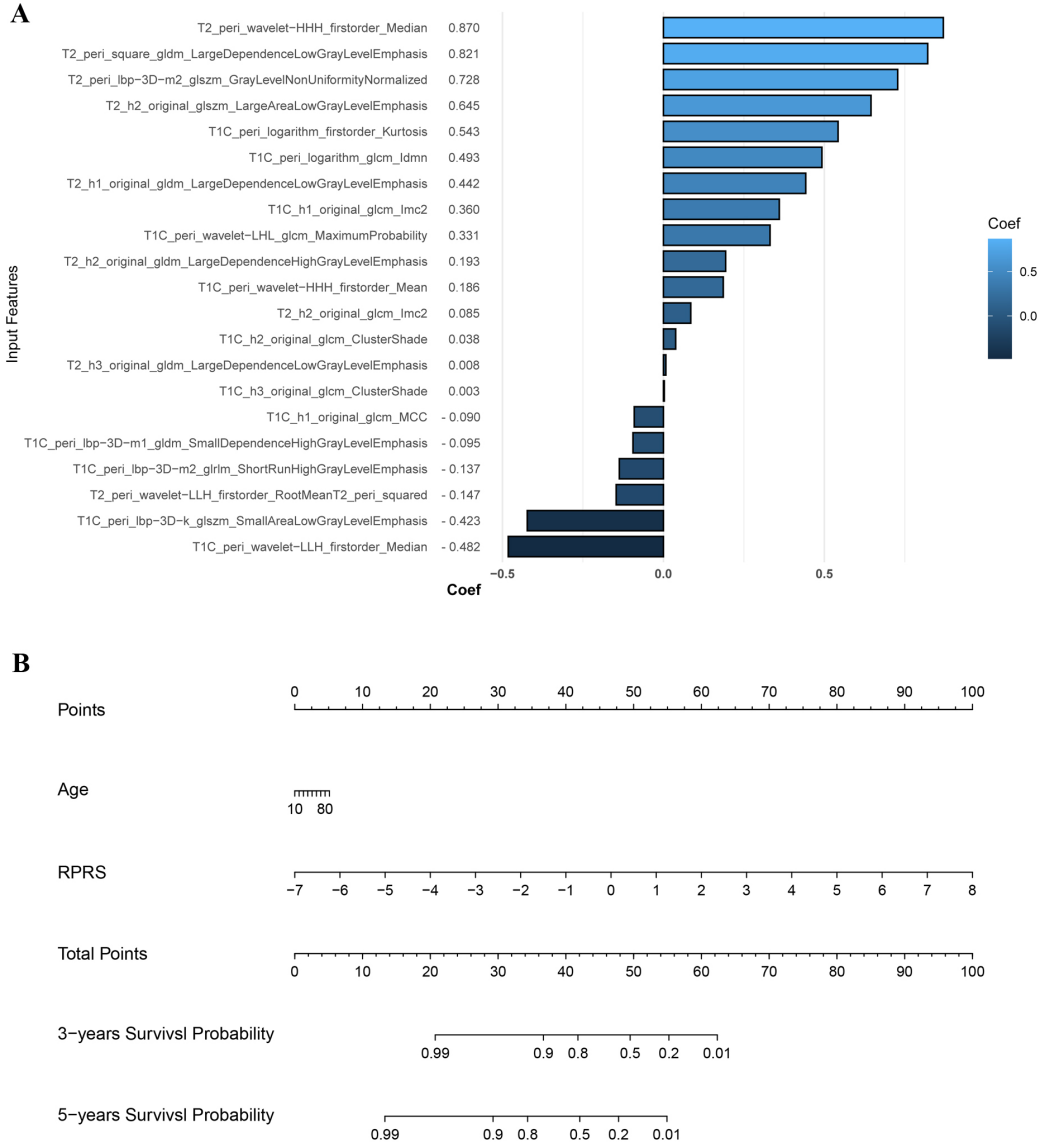
The input features and corresponding regression coefficients of radiomics progression risk score (RPRS) and the nomogram. **(A)** The features and corresponding coefficients for RPRS calculation. The feature with greatest predictive contribution was a wavelet transformed feature derived from the peritumor region on fat-suppressed T2-weighted imaging. **(B)** Nomogram for prediction of progression risk.

Baseline habitat features are shown in **Supplementary Table S4**. Nine baseline habitat features-based predictive signatures yielded unconvincing performance in the validation cohort (**Table 2**). The selected radiomics features in each predictive signature are shown in **Supplementary Table S5**. As shown in **Table 3**, among all the radiomics signatures, the Peri-tumor + Habitat _combined signature yielded relatively stable and excellent performance for prediction progression: in the training cohort, the C-index was 0.868 (95% confidence interval [CI], 0.809–0.927), median AUC was 0.914, and IBS was 0.091; in the validation cohort, the C-index was 0.761 (95% CI, 0.647–0.875), median AUC was 0.775, and IBS was 0.131. As a result, this signature was identified as the best performing radiomics signature and was entered into the follow-up study. The RPRS was calculated according to the input features and corresponding regression coefficients in the Peri-tumor + Habitat _combined signature (**Figure 3A**).

**Table 2:** Predictive performance of baseline habitat signatures.

Signature	Training cohort				Validation cohort			
	C-index	95%CI	AUC	IBS	C-index	95%CI	AUC	IBS
Voxel-number_1	0.548	0.451-0.645	0.526	0.191	0.510	0.341-0.680	0.521	0.182
Voxel-number_2	0.490	0.374-0.607	0.515	0.189	0.469	0.318-0.620	0.512	0.180
Voxel-number_3	0.525	0.408-0.643	0.562	0.189	0.480	0.316-0.645	0.470	0.179
Voxel-number_ combined	0.624	0.523-0.724	0.598	0.189	0.425	0.285-0.566	0.395	0.183
Voxel-fraction _1	0.591	0.496-0.687	0.585	0.188	0.494	0.352-0.637	0.527	0.182
Voxel-fraction _2	0.515	0.416-0.614	0.581	0.188	0.520	0.400-0.639	0.423	0.180
Voxel-fraction _3	0.576	0.480-0.672	0.608	0.192	0.517	0.378-0.656	0.500	0.178
Voxel-fraction _combined	0.592	0.496-0.689	0.580	0.188	0.499	0.364-0.634	0.439	0.181
Voxel_combined	0.602	0.500-0.705	0.601	0.188	0.448	0.300-0.597	0.412	0.182

95%CI, 95% confidence interval of C-index; AUC, median AUC of the time-dependent receiver operating characteristic curve; IBS, integrated Brier score.

**Table 3:** Predictive performance of conventional radiomics signatures and habitat radiomics signatures.

Signature	Training cohort				Validation cohort			
	C-index	95%CI	AUC	IBS	C-index	95%CI	AUC	IBS
Conventional radiomics signatures								
Tumor region	0.756	(0.673-0.840)	0.808	0.140	0.494	(0.346-0.643)	0.505	0.180
Peri-tumor	0.829	(0.757-0.901)	0.886	0.101	0.639	(0.502-0.776)	0.636	0.182
Tumor expansion	0.775	(0.698-0.852)	0.803	0.148	0.618	(0.531-0.705)	0.672	0.180
Tumor region + peri-tumor _combined	0.832	(0.757-0.907)	0.887	0.096	0.572	(0.459-0.686)	0.558	0.185
Habitat radiomics signatures								
Habitat1	0.707	(0.625-0.789)	0.734	0.158	0.609	(0.490-0.728)	0.629	0.181
Habitat2	0.699	(0.608-0.790)	0.717	0.175	0.554	(0.422-0.686)	0.603	0.180
Habitat3	0.675	(0.566-0.784)	0.733	0.173	0.547	(0.421-0.673)	0.544	0.181
Habitat _ combined	0.758	(0.677-0.838)	0.741	0.157	0.563	(0.449-0.677)	0.570	0.180
Peri-tumor + Habitat _combined	0.868	(0.809-0.927)	0.914	0.091	0.761	(0.647-0.875)	0.775	0.131

C-index, concordance index; 95%CI, 95% confidence interval of C-index; AUC, median AUC of the time-dependent receiver operating characteristic curve; IBS, integrated Brier score.

### Model construction and performance evaluation

Age was the only significant clinical prognostic predictor of progression in the univariable Cox regression analysis (**Supplementary Table S6**) on which the clinical model was based. The nomogram for individualized risk assessment integrating the RPRS and age is shown in **Figure 3B**.

**Figure 4:**
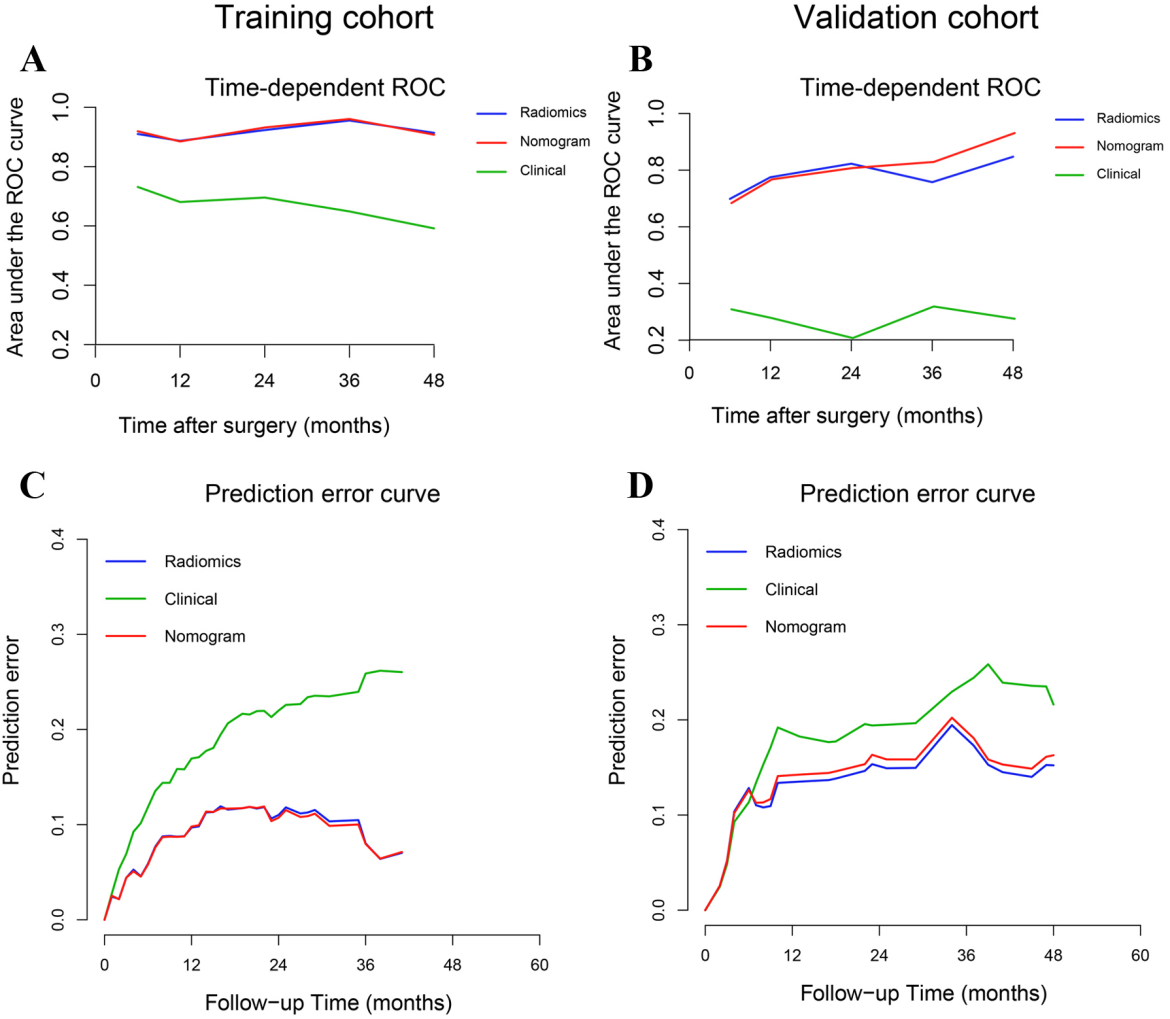
Time-dependent receiver operating characteristic curves and prediction error curves for the radiomics signature, nomogram, and clinical models in the training **(A, C)** and validation **(B, D)** cohorts.

**Figure 5:**
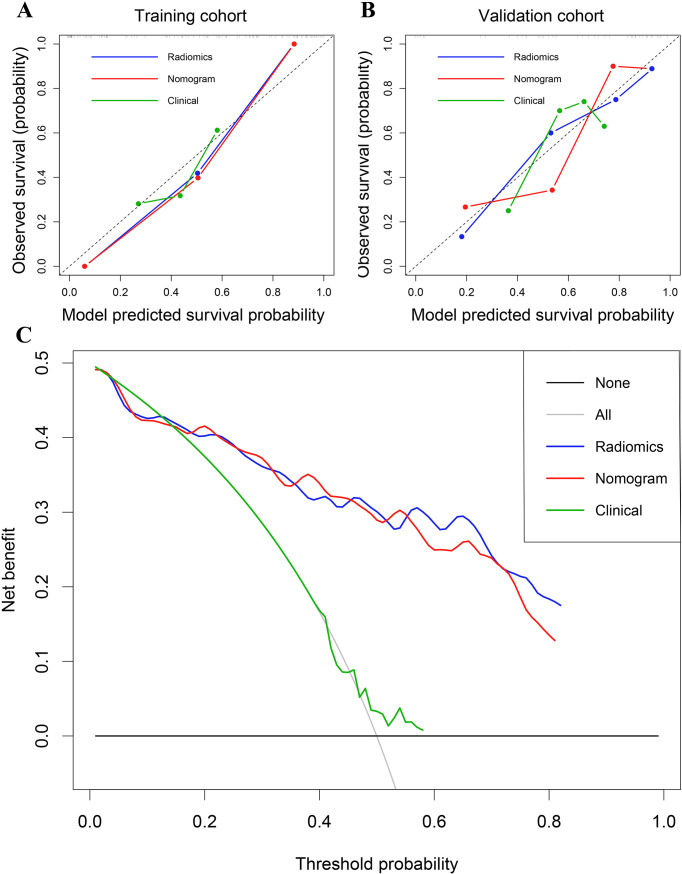
**(A)** Calibration curves of the radiomics signature, nomogram, and clinical models in the training cohort. **(B)** Calibration curves in the validation cohort. **(C)** Decision curve analysis for the entire cohort.

The predictive performance of the radiomics signature, clinical model, and nomogram is shown in **Table 4**. The C-index for prediction of progression in the training and validation cohorts was highest for the nomogram (0.874 [95% CI, 0.819–0.930] and 0.777 [95% CI, 0.660–0.894], respectively). In the validation cohort, the AUC was slightly higher for the nomogram (0.808) than the radiomics model (0.775, *P* = 0.005) and the clinical model (0.278, *P* = 0.293; **Figures 4A, B**). The predictive error of the models is shown in **Figures 4C, D**. In the validation cohort, the IBS for the nomogram was 0.135, which was lower than that of the clinical model (0.175); the IBS of the nomogram and radiomics model (0.131) were similar. Decision curve analysis of the nomogram showed a good clinical benefit within the full range of threshold probability (**Figure 5C**).

**Table 4:** Predictive performance of radiomics signature, clinical model, and nomogram.

Model	Training cohort					Validation cohort				
	C-index	95%CI	AUC	IBS	P	C-index	95%CI	AUC	IBS	P
Radiomics	0.868	0.809-0.927	0.923	0.091	0.145	0.761	0.647-0.875	0.775	0.131	0.293
Clinical	0.668	0.563-0.773	0.681	0.183	<0.001	0.336	0.212-0.459	0.278	0.175	0.005
Nomogram	0.874	0.819-0.930	0.919	0.090	ref	0.777	0.660-0.894	0.808	0.135	ref

C-index, concordance index; 95%CI, 95% confidence interval of C-index; AUC, median AUC of the time-dependent receiver operating characteristic curve; IBS, integrated Brier score.

### Progression risk stratification and survival analysis

**Figure 6:**
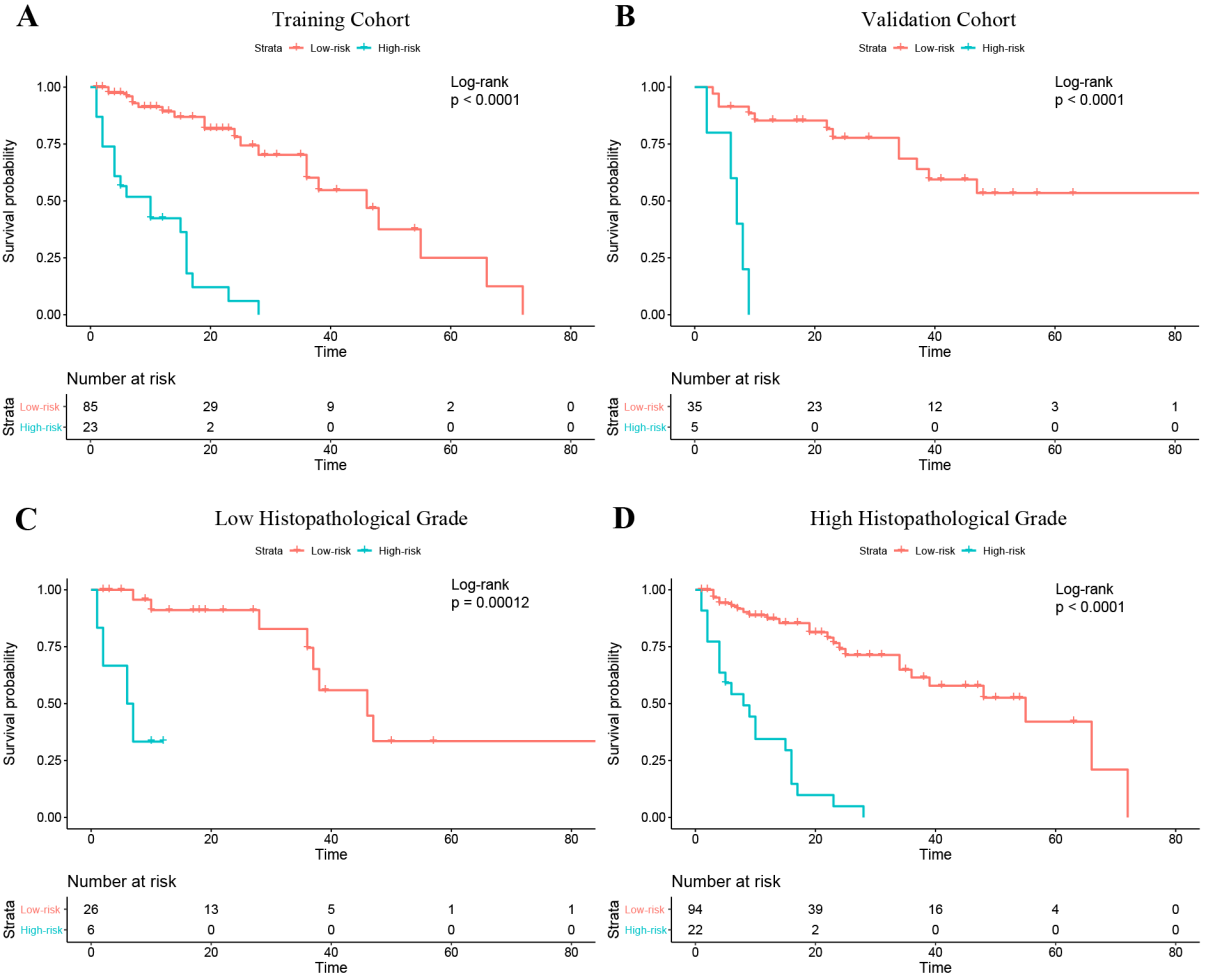
Kaplan–Meier curves of progression-free survival in the patients with low and high risk of progression based on the nomogram. **(A)** Training cohort. **(B)** Validation cohort. **(C)** The Low histopathological grade group of the entire cohort; **(D)** The high histopathological grade group of the entire cohort.

In the training cohort, the optimal cutoff for nomogram risk score to divide patients into two risk classifications was 1.28. Kaplan–Meier curves of patients in both the training and validation cohorts grouped according to risk of progression are shown in **Figures 6A, B**. PFS significantly differed between the groups in both cohorts (P<0.01). In addition, the nomogram could stratify patients in the overall cohort for PFS in both low and high histopathological grade subgroup (**Figures 6C, D**).

## Discussion

In this study, we verified that a radiomics model combining intratumoral habitat features and peritumor features can predict tumor progression in patients with STS. PFS in our cohort ranged from less than 1 month (5 patients) to over 5 years (7 patients). Compared with analyzing radiomics features derived from intratumoral habitats or regions, the peritumor region, or tumoral expansion, the combined radiomics features signature yielded better predictive performance. Moreover, in the validation cohort, the nomogram showed a convincing level of performance (C-index, 0.777), less prediction error (IBS ≤0.135), good calibration, and convincing clinical usefulness.

Conventionally, radiomics has focused on analyzing the primary tumor as a whole. However, in consideration of the inherent internal heterogeneity and peritumoral aggressiveness of the tumor, it is conceivable that subregions within the tumor and regions surrounding it contain complementary useful information (28). In a previous study, the survival prediction performance of integrated features was better with integrated features than with intra- and peritumoral features alone (22). Another study suggested that a radiomics model based on tumor region habitats enabled accurate patient risk stratification (18). In our study, intra- and peritumoral features were integrally analyzed to construct a survival prediction model, which achieved a convincing performance and revealed that comprehensive analysis of multi-regional and multi-scale radiomics information can quantify tumor heterogeneity. The integrated model appears to have considerable potential in prognostication of STS patients.

Empirical evidence has shown that the tumor microenvironment might have an indispensable role in STS tumor relapse (29). Morphologic changes in the microenvironment that influence survival can be detected by peritumoral radiomics and peritumoral radiomics has potential for predicting progression (28). Dou et al. (30) analyzed radiomics features derived from a 3 to 9 mm region outside the tumor margin to predict distant metastasis of lung adenocarcinoma. Other studies have suggested that radiomics based on a region 15 mm outside of the tumor can stratify patients according to prognosis and predict the response to neoadjuvant therapy (21, 22). In a study conducted by Braman et al. (21), the peritumoral radiomics features included in the final prediction signatures were all derived from the region within 12 mm of the tumor margin; no feature from beyond 12 mm was included. In our study, the region 15 mm outside the STS lesion contained a large amount of bone, large vessels and air; therefore, we defined the peritumoral region boundary as 10 mm from the tumor margin. The peritumoral signature yielded better performance than other single-region signatures, demonstrating that the peritumoral region contains important information regarding STS progression.

Considering the significant variability observed across intratumoral regions, image-based partitioning has been used to identify relevant subregions important for prediction of tumor biological behavior (15, 16). High-throughput radiomics features can be screened for constructing quantitative models for oncology diagnostics. Verma et al. manually partitioned subregions within glioblastomas on the basis of multi-sequence MRI and analyzed the radiomics features derived from each subregion to predict tumor progression (18). However, manual partitioning is reliant on radiologist experience and can only be applied in partitioning of contiguous subregions, which may result in poor reproducibility and objectivity. The clustering of voxels in multi-sequence MRI is a data-based analysis method that enables segmentation of subregions of similar tissue at a voxel-wise level (31). Previous studies have demonstrated that voxel number or fraction of cluster-segmented habitats in functional or structural MRI is an efficient biomarker for tumor biological behavior prediction (32, 33). Nevertheless, these studies focused on analyzing a small number of habitat baseline characteristics and neglected high-dimensional radiomics features that depict tumor habitat heterogeneity. In our study, we considered the potential of integrating high-throughput radiomics feature analysis and voxel-based habitat segmentation to predict STS progression. We showed that the combination of radiomics features derived from intratumoral cluster-segmented habitats and peritumoral features yielded the best predictive performance, validating that intratumoral habitat radiomics features at the voxel level adds predictive value.

Neoadjuvant radiotherapy treatment plays a dominant role in improving prognosis in STS patients (34). Hence, it is vital to identify patients with high risk of progression and treat them accordingly. Our study demonstrated that the radiomics nomogram, which integrated voxel-based and multiregional radiomics features with clinical information, yielded favorable performance for PFS prediction and provided convincing risk stratification ability. Our nomogram generated two risk stratifications (low or high risk of progression) and should help fellow clinicians with management of individual STS patients. For patients with a low risk of progression, surgery without adjuvant therapy might be considered initially to avoid the side effects of chemoradiotherapy. For those with a high risk, postoperative systemic adjuvant chemoradiotherapy and targeted therapy should be considered. In current clinical practice, the most important prognostic indicator for STS is histopathological grade (35). According to our study, the stratification ability of the nomogram was further proved by the sub-cohort analysis in the low- and high-grade patients defined by histopathological grade system. Thus, use of our nomogram can provide incremental information to clinicians and STS patients and help guide treatment decisions.

Several study limitations should be mentioned. First, owing to its retrospective design, selection bias was probably present. Second, the radiomics generalizability and robustness across inconsistent MRI parameters and multiple institutions should be validated. Although we used standardization processes at the imaging and feature levels, more prospective data is needed to validate our findings. Finally, tumor boundaries were defined manually (first outlined by a junior radiologist and corrected by a senior one). Semi-automatic or automatic delineation should be used in future studies to minimize delineator bias.

In conclusion, we constructed a nomogram based on intratumoral habitat and peritumor radiomics that predicts tumor progression in STS patients and stratifies them according to risk of progression. Performance of the nomogram was superior to that of other habitat- and radiomics-based models.

## Data Availability

The original contributions presented in the study are included in the article/**Supplementary Material**. Further inquiries can be directed to the corresponding author/s.
