# Supplementary material for: Intratumoral habitat and peritumor radiomics for progression risk stratification of patients with soft tissue sarcoma: a multicenter study
Source: Front Oncol. 2026 Jan 19;15:1619704. doi: 10.3389/fonc.2025.1619704 (PMC12861874; doi:10.3389/fonc.2025.1619704)
Supplement: Supplementary file 1 [file Table1.docx]

**Supplementary material**

**Supplement A1. Clinical baseline information, postoperative histopathological indicators and semantic MRI features**

Clinical baseline information contained age and gender. Postoperative histopathological indicators contained 4 pathological grading and staging. The FNCLCC and NCI grading systems were used. Staging was performed using the AJCC system (8th version). FNCLCC grade I was defined as histopathological low grade; grades II and III were defined as histopathological high grade. Two radiological experts (Dr. Gao and Dr. Wang)with more than 7 years of experience in musculoskeletal diagnosis evaluated semantic MRI features, which comprised the following: (1) number (solitary or multiple); (2) depth (deep or superficial; maximum lesion depth ≥8 cm was defined as deep); (3) heterogeneous signal intensity on FS-T2WI (<50% or ≥50%); (4) tumor volume exhibiting necrotic MRI signal (none, 1%–50%, or ≥50% of tumor volume); (5) peritumoral edema (none, limited, or extensive); and (6) location (limb, trunk wall, head and neck, or internal trunk wall).

**Supplement A2. Image preprocessing and segmentation of tumor-associated regions**

First, registration of FS-T2WI and CE-T1WI images from each patient was performed using three-dimensional rigid transformation with 3D-slicer software version 5.0.3 (www. slicer.org). Next, we applied the N4-bias-field-correction module in Python to correct the bias field inhomogeneity of each image. Then, using ITK-SNAP software version 3.8.0 (www.itksnap.org), three-dimensional tumoral regions of interest were manually delineated by the junior radiologist (Dr. Liang) and corrected by the senior radiological experts (Dr. Wang) to form the tumor region mask. Radiomics Intelligent Analysis Software was then used to generate the peritumoral and tumoral expansion masks of each lesion using morphologic dilation at a voxel value of 10 mm outside the tumor lesion mask. Uninvaded bone tissue, large vessels and air areas in both peritumoral mask and tumoral expansion mask were manually wiped out. Finally, all images and masks were resampled at an isotropic spatial voxel size of 1 × 1 × 1 mm^3^ via Radiomics Intelligent Analysis Software.

**Supplement A3. Survival signatures determination**

Univariate Cox regression analysis was first applied for progression-related factors filtration (threshold, P <0.05). Then, the minimum redundancy maximum relevance algorithm was used to screen the top 30 features correlated with progression. Finally, using the least absolute shrinkage and selection operator Cox regression algorithm, further screening of the feature parameters was performed and a set of progression predictive radiomics signatures was constructed.

**Supplement A4. Survival model construction**

The clinical baseline information, postoperative histopathological indicators and semantic MRI features that demonstrated statistical significance in univariate Cox regression analyses were entered into multivariate analysis. Those with *P* <0.05 in the multivariate Cox regression analysis were determined as independent prognostic predictors and used for clinical model construction. Accordingly, a radiomics nomogram combining the independent prognostic predictors and the best performing radiomics signature was constructed.

Table S1. Histopathological confirmation

|  | No. of patients | |
| --- | --- | --- |
|  | Training cohort  (n=108) | Validation cohort  (n=40) |
| Dedifferentiated liposarcoma | 7 | 1 |
| Myxoid liposarcoma | 17 | 7 |
| Myxofibrosarcoma | 10 | 2 |
| Synovial sarcoma | 5 | 4 |
| Pleomorphic liposarcoma | 3 | 2 |
| Undifferentiated pleomorphic sarcoma | 17 | 1 |
| Malignant peripheral nerve sheath tumor | 1 | 2 |
| Leiomyosarcoma | 7 | 3 |
| Angiosarcoma | 4 | 0 |
| Embryonal rhabdomyosarcoma | 0 | 1 |
| Pleomorphic rhabdomyosarcoma | 1 | 0 |
| Spindle cell rhabdomyosarcoma | 0 | 4 |
| Extraskeletal osteosarcoma | 4 | 0 |
| Epithelioid sarcoma | 0 | 1 |
| Alveolar soft part sarcoma | 3 | 1 |
| Spindle cell sarcoma, undifferentiated | 1 | 4 |
| Solitary fibrous tumor, malignant | 12 | 3 |
| Undifferentiated sarcoma | 8 | 2 |
| Others | 8 | 2 |

Table S2. MRI acquisition parameters

|  | TR  (ms) | TE  (ms) | Slice Thickness  (mm) | Slice Spacing  (mm) | Matrix | Field of View |
| --- | --- | --- | --- | --- | --- | --- |
| FS-T2WI | 2400–4500 | 70–120 | 3–5 | 1 | 320 × 320 | 200-400 |
| CE-T1WI | 500–600 | 10–15 | 3–5 | 1 | 320 × 320 | 200-400 |

Note: TR, time of repetition; TE, time of echo; FS-T2WI, fat-suppressed T2 weighted imaging; CE-T1WI, contrast enhanced fat-suppressed T1 weighted imaging

Table S3. Predictive signatures and enrolled features

| Signature | Enrolled features |
| --- | --- |
| Conventional radiomics signature set | |
| Tumor region | tumor region radiomics features |
| Peri-tumor | peri-tumor radiomics features |
| Tumor expansion | tumor expansion radiomics features |
| Tumor region + peri-tumor _combined | tumor region radiomics features,  peri-tumor radiomics features |
| Habitat baseline signature set | |
| Voxel-number_1 | habitat1 voxel number |
| Voxel-number_2 | habitat2 voxel number |
| Voxel-number_3 | habitat3 voxel number |
| Voxel-number_ combined | habitat1 voxel number,  habitat2 voxel number,  habitat3 voxel number |
| Voxel-fraction _1 | habitat1 voxel fraction |
| Voxel-fraction _2 | habitat2 voxel fraction |
| Voxel-fraction _3 | habitat3 voxel fraction |
| Voxel-fraction _combined | habitat1 voxel fraction,  habitat2 voxel fraction,  habitat3 voxel fraction |
| Voxel _combined | habitat1 voxel number,  habitat2 voxel number,  habitat3 voxel number,  habitat1 voxel fraction,  habitat2 voxel fraction,  habitat3 voxel fraction |
| Habitat radiomics signature set | |
| Habitat1 | habitat1 radiomics features |
| Habitat2 | habitat2 radiomics features |
| Habitat3 | habitat3 radiomics features |
| Habitat _ combined | habitat1 radiomics features,  habitat2 radiomics features,  habitat3 radiomics features |
| Peri-tumor + Habitat _combined | habitat1 radiomics features,  habitat2 radiomics features,  habitat3 radiomics features,  peri-tumor radiomics features |

Table S4. Baseline habitat features

|  |  | Training cohort | Validation cohort | P |
| --- | --- | --- | --- | --- |
| Voxel number | Habitat 1 | 3312 (509, 34158) | 6183 (714, 96788) | 0.212 |
|  | Habitat 2 | 37829 (13567, 127444) | 44828 (12123, 123766) | 0.839 |
|  | Habitat 3 | 39170 (11555, 173790) | 58287 (24027, 327801) | 0.108 |
| Voxel fraction | Habitat 1 | 0.030 (0.009, 0.139) | 0.042 (0.012, 0.148) | 0.442 |
|  | Habitat 2 | 0.485 (0.164, 0.728) | 0.382 (0.096, 0.649) | 0.144 |
|  | Habitat 3 | 0.420 (0.173, 0.602) | 0.448 (0.309, 0.656) | 0.148 |

Note: Data are median (inter-quartile range)

Table S5. Input features in conventional radiomics signatures and habitat radiomics signatures

| Signature | Input features |
| --- | --- |
| Tumor region | T1C_reg_lbp.3D.m2_glcm_SumEntropy;  T1C_reg_lbp.3D.k_glszm_HighGrayLevelZoneEmphasis;  T1C_reg_log.sigma.5.0.mm.3D_firstorder_Skewness;  T1C_reg_wavelet.LHL_glrlm_RunVariance;  T1C_reg_log.sigma.5.0.mm.3D_glcm_ClusterShade;  T1C_reg_wavelet.LLH_glcm_ClusterShade;  T1C_reg_wavelet.LLL_glcm_ClusterShade;  T1C_reg_wavelet.HHL_glcm_ClusterProminence;  T2_reg_exponential_glcm_Imc2;  T1C_reg_log.sigma.3.0.mm.3D_gldm_LargeDependenceHighGrayLevelEmphasis;  T1C_reg_original_firstorder_Minimum;  T1C_reg_lbp.3D.k_glszm_SizeZoneNonUniformityNormalized;  T2_reg_lbp.3D.k_glcm_DifferenceEntropy |
| Peri-tumor | T1C_peri_logarithm_firstorder_Skewness;  T1C_peri_lbp.3D.m1_gldm_SmallDependenceHighGrayLevelEmphasis;  T1C_peri_square_glcm_Correlation;  T1C_peri_wavelet.LHL_glszm_LargeAreaHighGrayLevelEmphasis;  T2_peri_wavelet.LLH_firstorder_Median;  T1C_peri_wavelet.HHH_firstorder_Mean;  T2_peri_wavelet.HHH_firstorder_Median;  T2_peri_squareroot_glcm_ClusterShade;  T2_peri_wavelet.LLH_glcm_DifferenceVariance;  T2_peri_square_gldm_LargeDependenceLowGrayLevelEmphasis;  T1C_peri_logarithm_firstorder_Kurtosis;  T1C_peri_wavelet.LLH_firstorder_Median;  T2_peri_lbp.3D.m2_glszm_GrayLevelNonUniformityNormalized;  T1C_peri_wavelet.LHL_glcm_MaximumProbability;  T1C_peri_lbp.3D.k_glszm_SmallAreaLowGrayLevelEmphasis |
| Tumor expansion | T1C_exp_squareroot_glcm_Idmn;  T1C_exp_squareroot_glszm_ZonePercentage;  T1C_exp_wavelet.HLL_glcm_ClusterShade;  T2_exp_original_glszm_LargeAreaHighGrayLevelEmphasis;  T1C_exp_logarithm_firstorder_Kurtosis;  T1C_exp_wavelet.LHL_glszm_LargeAreaHighGrayLevelEmphasis;  T2_exp_square_firstorder_Median;  T1C_exp_gradient_firstorder_Skewness;  T2_exp_wavelet.LLH_firstorder_Median;  T1C_exp_wavelet.HHH_firstorder_Mean;  T2_exp_exponential_glcm_Idmn;  T1C_exp_wavelet.LHL_glcm_MaximumProbability;  T2_exp_squareroot_glcm_DifferenceEntropy;  T2_exp_lbp.3D.m1_firstorder_Skewness |
| Tumor region + peri-tumor _combined | T1C_peri_lbp.3D.m1_gldm_SmallDependenceHighGrayLevelEmphasis;  T1C_peri_exponential_glrlm_ShortRunLowGrayLevelEmphasis;  T1C_peri_wavelet.LHL_glszm_LargeAreaHighGrayLevelEmphasis;  T2_peri_wavelet.HHH_firstorder_Median;  T1C_reg_squareroot_glrlm_GrayLevelNonUniformityNormalized;  T2_reg_lbp.3D.k_glcm_Idmn;  T2_peri_squareroot_glcm_ClusterShade;  T1C_peri_logarithm_firstorder_Kurtosis;  T2_peri_wavelet.LLH_glcm_DifferenceVariance;  T1C_reg_wavelet.LHL_firstorder_Skewness;  T2_peri_square_gldm_LargeDependenceLowGrayLevelEmphasis;  T1C_reg_wavelet.LLL_gldm_LowGrayLevelEmphasis;  T1C_peri_wavelet.LLH_firstorder_Median;  T1C_reg_lbp.3D.k_glszm_HighGrayLevelZoneEmphasis;  T1C_peri_exponential_glcm_ClusterProminence;  T1C_peri_lbp.3D.k_glszm_SmallAreaLowGrayLevelEmphasis;  T1C_reg_lbp.3D.k_glszm_SizeZoneNonUniformityNormalized;  T2_peri_lbp.3D.m2_glszm_GrayLevelNonUniformityNormalized |
| Habitat1 | T1C_h1_original_glcm_ClusterShade;  T1C_h1_original_glcm_Id;  T1C_h1_original_glcm_Imc2;  T1C_h1_original_glcm_InverseVariance;  T1C_h1_original_glcm_MCC;  T1C_h1_original_ngtdm_Complexity;  T2_h1_original_gldm_LargeDependenceLowGrayLevelEmphasis |
| Habitat2 | T1C_h2_original_firstorder_Skewness;  T1C_h2_original_glcm_ClusterShade;  T2_h2_original_glcm_Imc2;  T2_h2_original_gldm_LargeDependenceHighGrayLevelEmphasis;  T2_h2_original_glszm_LargeAreaHighGrayLevelEmphasis |
| Habitat3 | T1C_h3_original_glcm_ClusterShade;  T1C_h3_original_glcm_Imc1;  T1C_h3_original_glcm_MaximumProbability;  T1C_h3_original_glrlm_RunLengthNonUniformityNormalized;  T2_h3_original_glcm_Imc1;  T2_h3_original_gldm_LargeDependenceLowGrayLevelEmphasis |
| Habitat _ combined | T1C_h3_original_glcm_MaximumProbability;  T2_h2_original_glszm_LargeAreaHighGrayLevelEmphasis;  T2_h2_original_glcm_Imc2;  T1C_h1_original_glcm_MCC;  T1C_h1_original_glcm_ClusterShade;  T1C_h2_original_firstorder_Skewness;  T2_h2_original_gldm_LargeDependenceHighGrayLevelEmphasis;  T2_h3_original_gldm_LargeDependenceLowGrayLevelEmphasis;  T1C_h2_original_glcm_ClusterShade;  T2_h1_original_gldm_LargeDependenceLowGrayLevelEmphasis |
| Peri-tumor + Habitat _combined | T1C_peri_wavelet-HHH_firstorder_Mean;  T1C_peri_lbp-3D-m1_gldm_SmallDependenceHighGrayLevelEmphasis;  T2_h2_original_gldm_LargeDependenceHighGrayLevelEmphasis;  T1C_peri_logarithm_firstorder_Kurtosis;  T2_h3_original_gldm_LargeDependenceLowGrayLevelEmphasis;  T1C_h1_original_glcm_MCC;  T2_peri_wavelet-HHH_firstorder_Median;  T1C_h3_original_glcm_ClusterShade;  T1C_peri_lbp-3D-m2_glrlm_ShortRunHighGrayLevelEmphasis;  T2_peri_wavelet-LLH_firstorder_RootMeanT2_peri_squared;  T2_h2_original_glcm_Imc2;  T1C_peri_wavelet-LLH_firstorder_Median;  T1C_peri_logarithm_glcm_Idmn;  T2_peri_square_gldm_LargeDependenceLowGrayLevelEmphasis;  T1C_h2_original_glcm_ClusterShade;  T2_h2_original_glszm_LargeAreaLowGrayLevelEmphasis;  T2_h1_original_gldm_LargeDependenceLowGrayLevelEmphasis;  T1C_peri_lbp-3D-k_glszm_SmallAreaLowGrayLevelEmphasis;  T1C_peri_wavelet-LHL_glcm_MaximumProbability;  T1C_h1_original_glcm_Imc2;  T2_peri_lbp-3D-m2_glszm_GrayLevelNonUniformityNormalized |

Table S6. Univariable Cox regression analysis of clinical, histopathological, and MRI Features

|  | HR | 95%CI | P |
| --- | --- | --- | --- |
| Clinical baseline information | | | |
| Age | 1.027 | 1.007-1.047 | 0.007* |
| Gender | 0.803 | 0.422-1.527 | 0.503 |
| Postoperative histopathological indicators | | | |
| FNCLCC | 1.549 | 0.923-2.598 | 0.098 |
| NCI | 1.082 | 0.694-1.686 | 0.729 |
| AJCC | 1.076 | 0.765-1.514 | 0.672 |
| Grade | 1.144 | 0.475-2.757 | 0.764 |
| Semantic MRI features | | | |
| Number | 1.179 | 0.594-2.341 | 0.637 |
| Depth | 0.775 | 0.397-1.515 | 0.456 |
| Heterogeneous SI at FS-T2WI | 0.926 | 0.491-1.749 | 0.814 |
| Tumor volume with MRI signal compatible with necrosis | 0.896 | 0.543-1.478 | 0.666 |
| Margin definitions at CE-T1WI | 0.766 | 0.448-1.311 | 0.331 |
| Peritumoral edema | 1.162 | 0.649-2.079 | 0.614 |
| Peritumoral enhancement | 1.101 | 0.573-2.116 | 0.772 |
| T-stage | 1.069 | 0.807-1.417 | 0.640 |
| N-stage | 1.158 | 0.531-2.523 | 0.712 |
| M-stage | 1.259 | 0.549-2.889 | 0.586 |
| Surgical margins | 1.257 | 0.608-2.599 | 0.537 |
| Radiotherapy | 1.124 | 0.589-2.145 | 0.723 |
| Chemotherapy | 0.787 | 0.392-1.581 | 0.501 |
| Location | 1.299 | 0.942-1.793 | 0.111 |

Note: HR, Hazard Ratio; 95%CI, 95% confidence interval of HR; FNCLCC, Fédération Nationale des Centres de Lutte Contre le Cancer; NCI, United States National Cancer Institute AJCC, American Joint Committee on Cancer. *, P < 0.05.
